# Protein and Skeletal Muscle Metabolism

**DOI:** 10.3390/nu18081218

**Published:** 2026-04-13

**Authors:** Donny Camera

**Affiliations:** Department of Biomedical, Health and Exercise Sciences, Swinburne University, Melbourne, VIC 3122, Australia; dcamera@swin.edu.au

Whole-body skeletal muscle mass is determined by the balance of two concurrent processes—muscle protein synthesis (MPS) and muscle protein breakdown (MPB)—collectively termed muscle protein turnover [1,2]. To promote muscle growth (hypertrophy), MPS must chronically exceed MPB on average to obtain a positive net protein balance. MPS is greatly affected by protein intake, which is in turn determined by the quantity and quality of constituent amino acids [3]. A current focus on nutrition-based interventions to promote skeletal muscle adaptation responses, particularly in combination with exercise, has centred on exploring how protein quality, intake adequacy, and systemic context (including clinical care and gut physiology) collectively determine muscle health across the lifespan. The recently published papers within this ‘Protein and Skeletal Muscle Metabolism’ Special Issue collectively highlight both progress and persistent gaps in our understanding of how to best optimise muscle protein anabolism and prevent sarcopenia.

A key mechanistic contribution comes from Ely et al. (2025) (contribution 1), who compared leucine-enriched β-lactoglobulin (BLG) with isonitrogenous whey protein isolate (WPI) in young healthy males. While BLG elicited higher circulating essential amino acid (EAA), branched-chain amino acid (BCAA), and leucine concentrations, both protein sources stimulated muscle protein synthesis (MPS) to a similar extent under feeding and feeding-plus-exercise conditions. This finding reinforces the concept of a “leucine threshold”, whereby, once a sufficient leucine level is reached, additional increases may not further augment MPS in healthy young individuals [4]. However, the enhanced aminoacidemia observed with BLG raises important translational questions: could leucine-enriched proteins confer advantages in populations with anabolic resistance, such as older adults or clinical patients? The study appropriately identifies this as a priority for future research.

In contrast to controlled experimental settings, Pérez-López et al. (2025) (contribution 2) provide real-world clinical evidence linking inadequate dietary intake to sarcopenia in patients with disease-related malnutrition. Their findings demonstrate that individuals consuming less than 70% of their energy and protein requirements exhibit significantly higher rates of sarcopenia and severe malnutrition. Notably, body mass index did not differ between sarcopenic and non-sarcopenic patients, underscoring the limitations of BMI as a diagnostic tool in this context. This study reinforces a critical clinical message: absolute intake adequacy—not merely protein quality—is a primary determinant of muscle preservation in at-risk populations. It also highlights the importance of routine dietary assessment and individualised nutritional support in clinical practice.

Bridging research and practice, Netzer et al. (2025) (contribution 3) reveal substantial gaps in the clinical competency of physical therapists in managing malnutrition–sarcopenia syndrome. Despite moderate awareness, the knowledge of diagnostic frameworks (e.g., EWGSOP2, GLIM) and practical skills in screening and treatment were limited. Given that MSS is both preventable and reversible, this competency gap represents a missed opportunity for early intervention. The study underscores the need for interdisciplinary education, clearer role delineation, and the integration of nutrition-focused training within rehabilitation curricula. Importantly, improving healthcare provider competence may be as impactful as advancing nutritional science itself, particularly in aging populations.

Expanding beyond traditional protein paradigms, Al-Refai et al. (2025) (contribution 4) introduce the microbiota–gut–muscle axis as a potential mediator of diet-induced effects on muscle metabolism. Differences in fibre content, anti-nutritional factors, and protein sources across vegan, vegetarian, and omnivorous diets may alter gut microbiota composition, which in turn could influence muscle anabolic signalling. While this conceptual framework is compelling, empirical evidence directly linking microbiota changes to functional muscle outcomes remains limited. This represents a significant knowledge gap: understanding whether—and how—diet-induced microbial shifts translate into meaningful differences in muscle mass and function.

Similarly, Yimam et al. (2025) (contribution 5) address the growing interest in sustainable and alternative protein sources. Their review highlights that plant-based and novel proteins often produce lower and slower postprandial aminoacidemia compared with animal-derived proteins yet may still achieve comparable anabolic outcomes. This apparent paradox challenges traditional assumptions that rapid and high amino acid availability is required for maximal MPS [5]. It suggests that factors such as protein digestibility, amino acid composition, feeding patterns, and possibly gut-mediated mechanisms may compensate for differences in aminoacidemia. However, variability across studies and protein sources indicates that not all alternatives are equivalent, emphasising the need for standardised evaluation frameworks.

Based on research presented in this Special Issue, future research should therefore prioritise (1) randomised controlled trials of leucine-enriched and alternative proteins in older and clinical populations; (2) integrated studies combining dietary assessment, muscle outcomes, and microbiome profiling to elucidate gut–muscle interactions; (3) comparative effectiveness research on sustainable protein sources, incorporating both metabolic and functional endpoints; and (4) implementation science approaches to improve the clinical screening, diagnosis, and management of malnutrition and sarcopenia.
